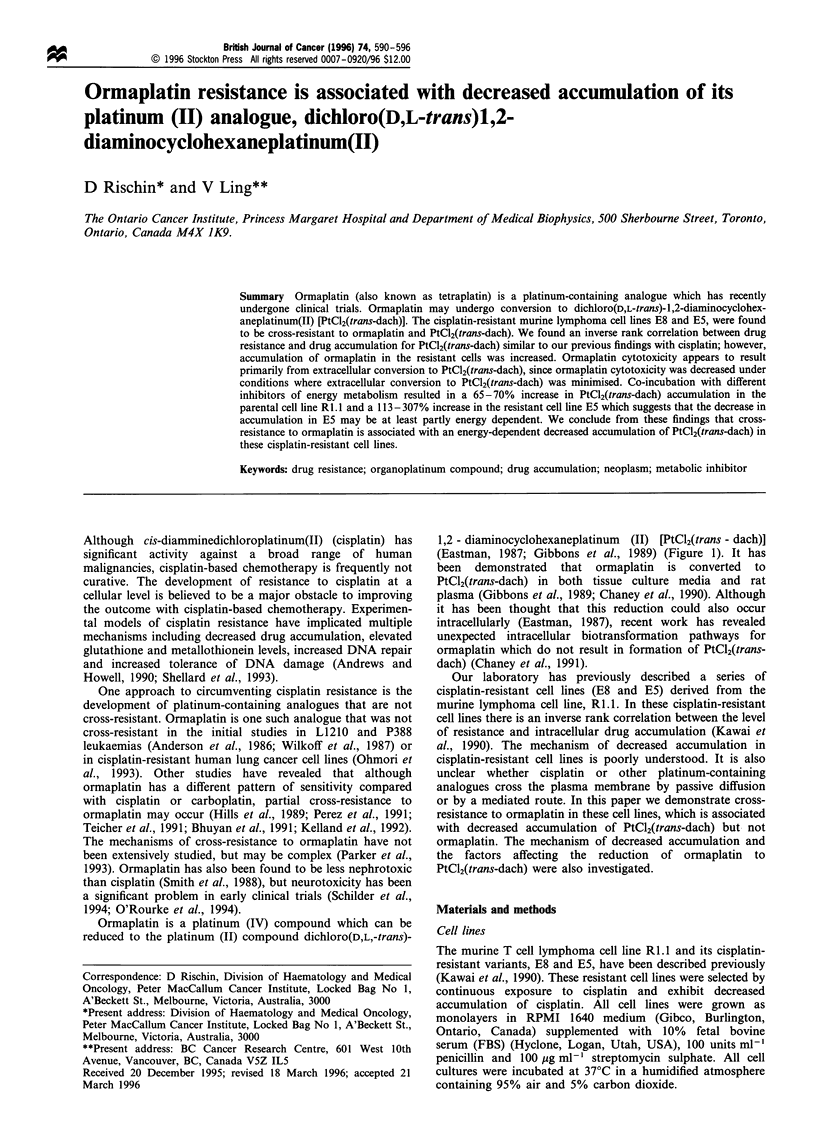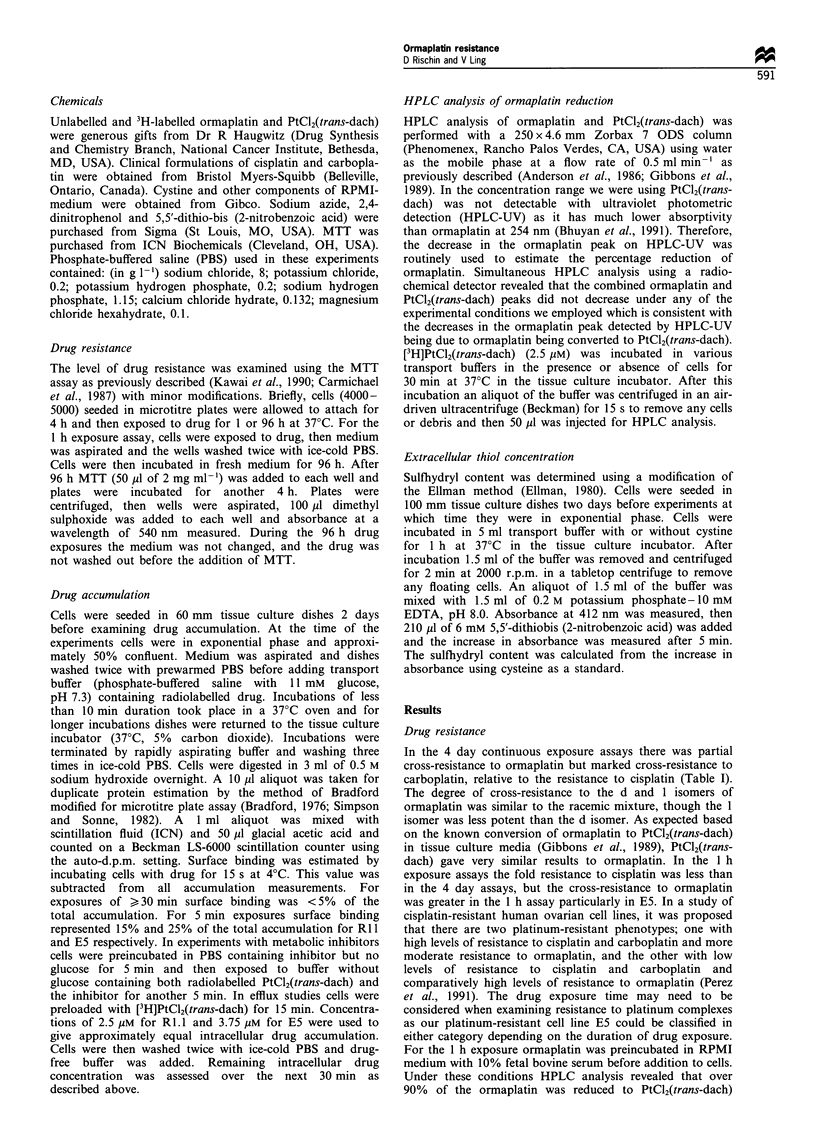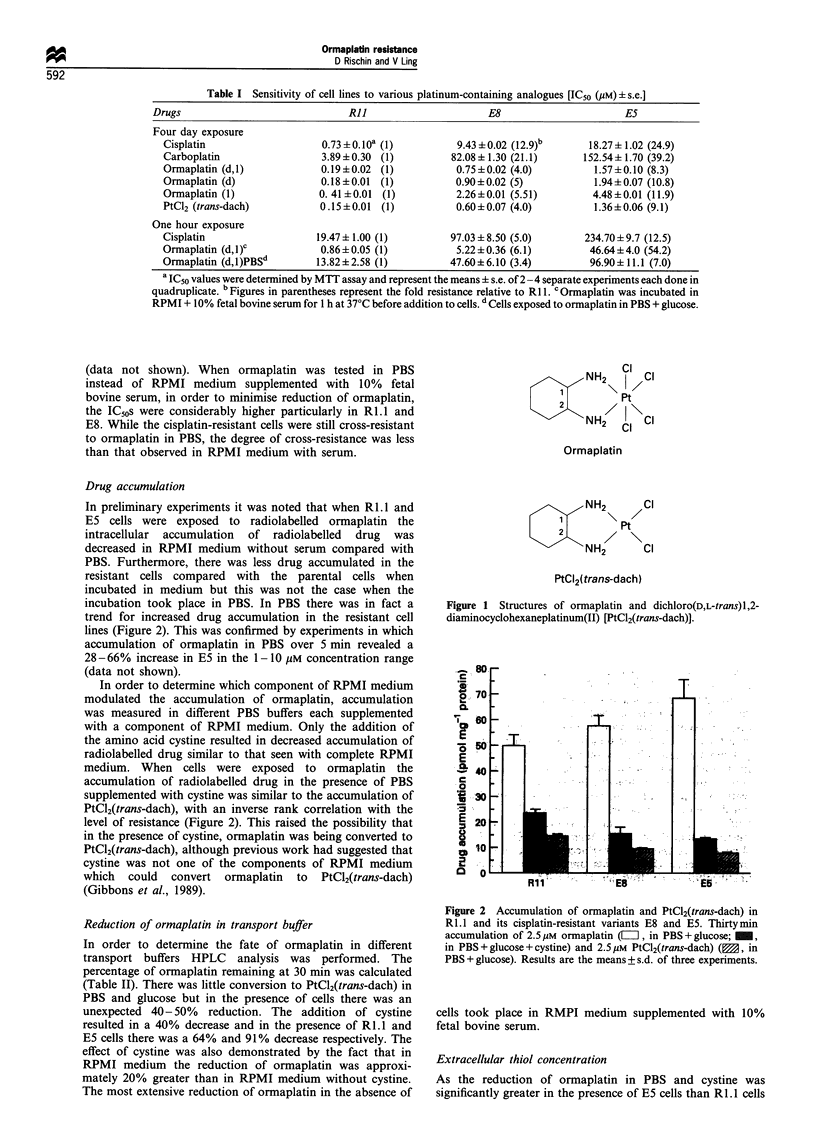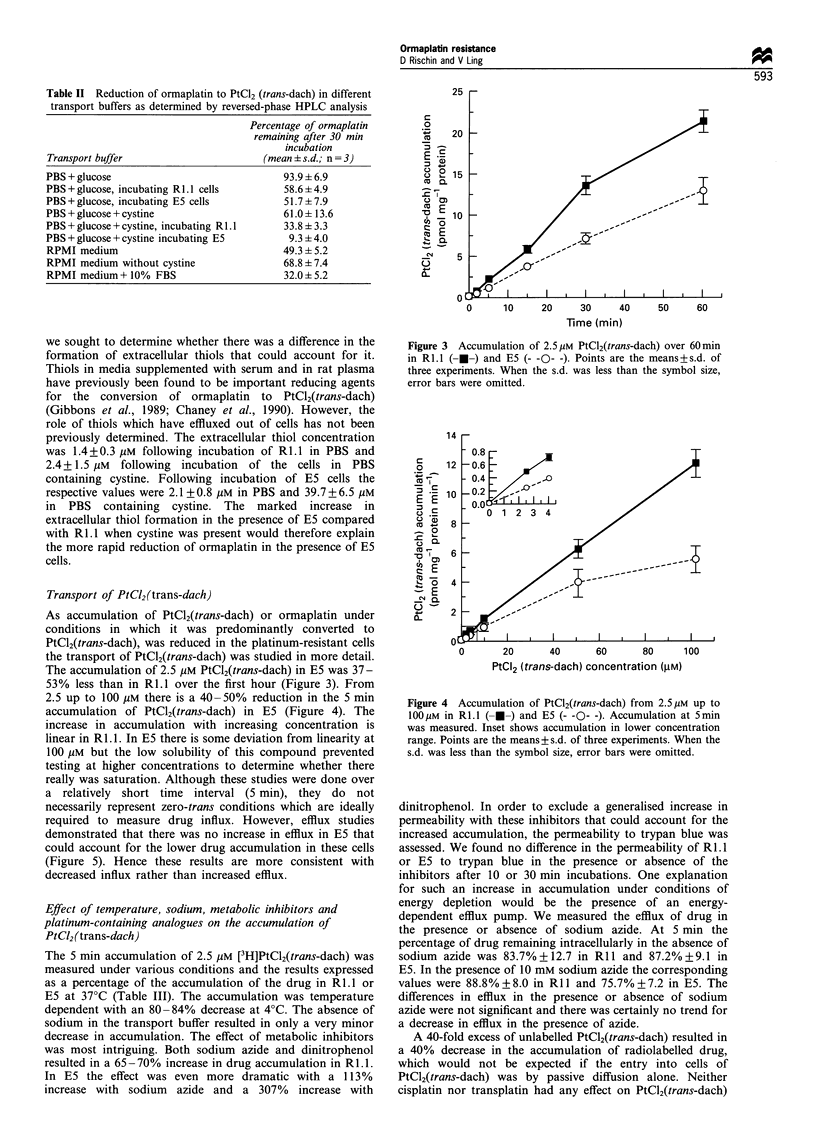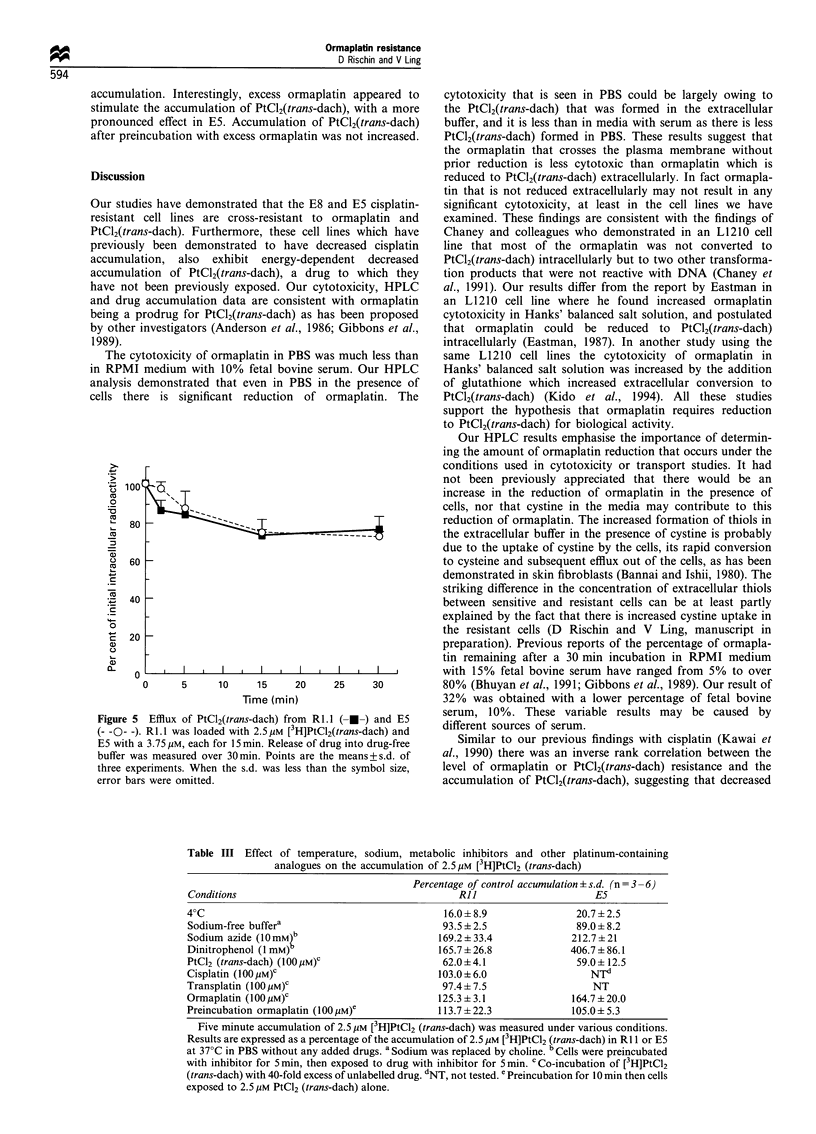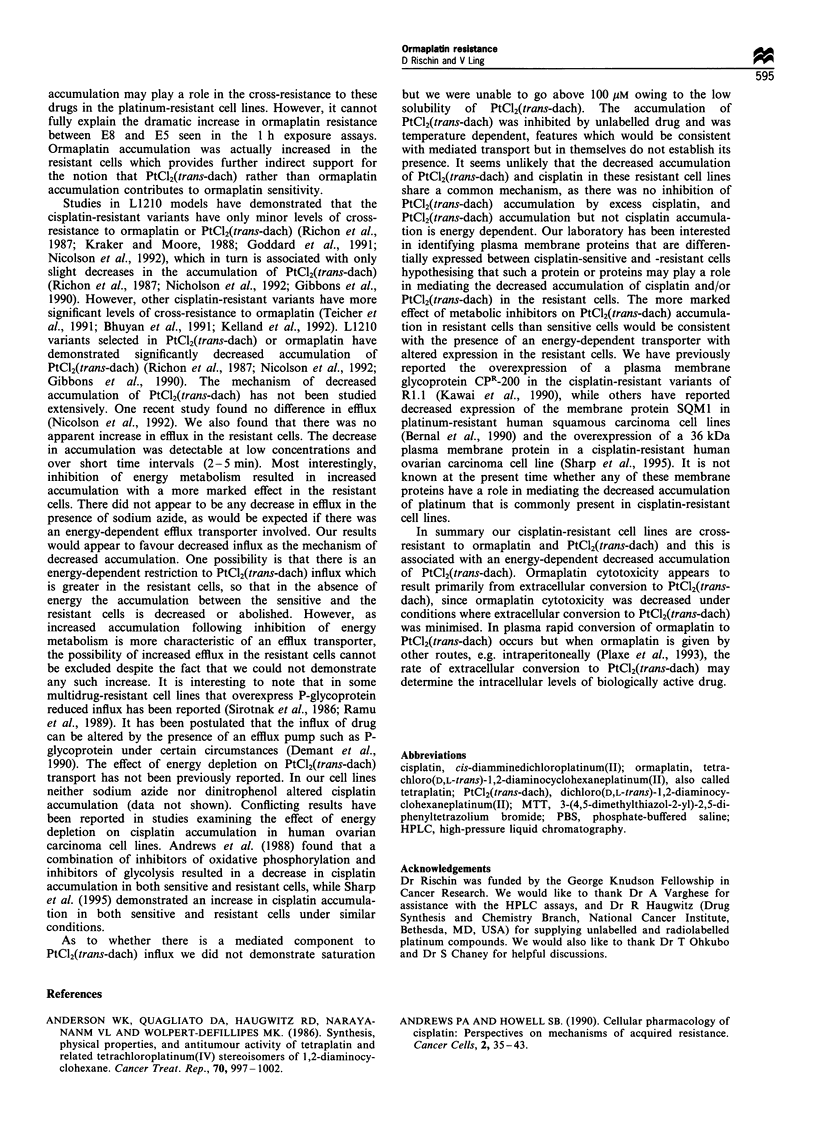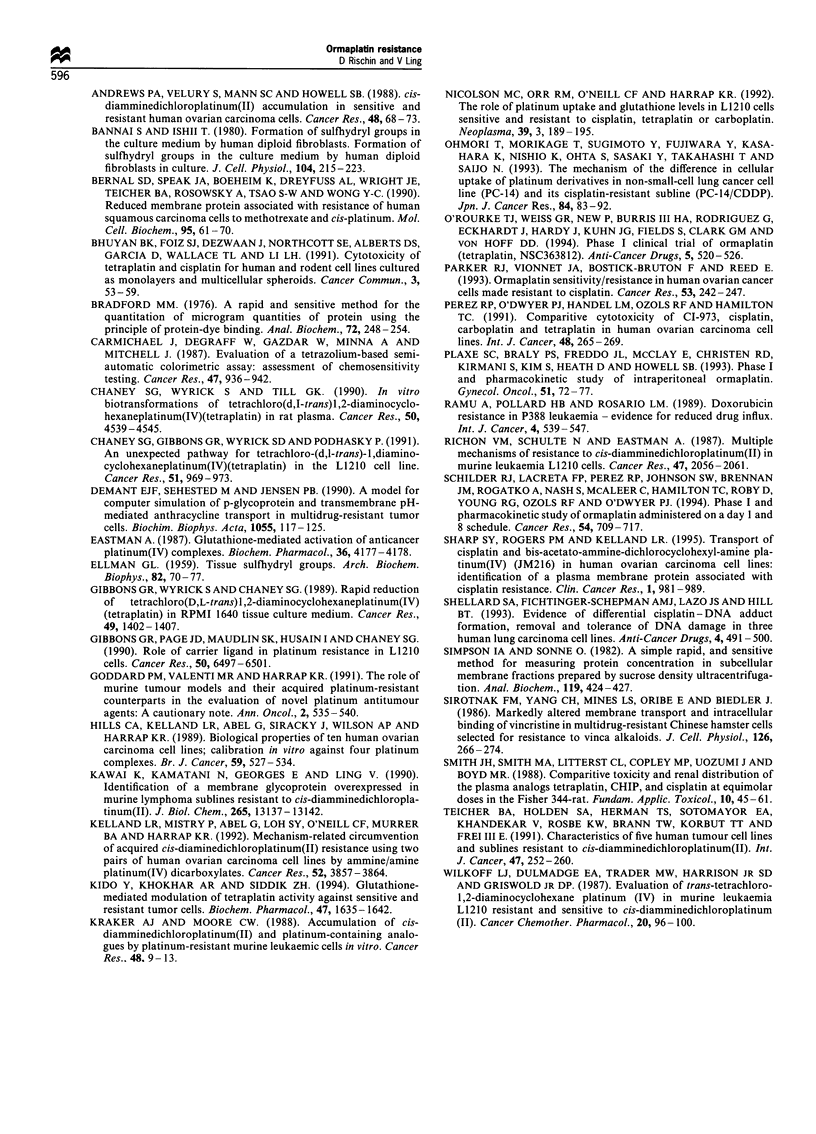# Ormaplatin resistance is associated with decreased accumulation of its platinum (II) analogue, dichloro(D,L-trans)1,2-diaminocyclohexaneplatinum (II).

**DOI:** 10.1038/bjc.1996.406

**Published:** 1996-08

**Authors:** D. Rischin, V. Ling

**Affiliations:** Ontario Cancer Institute, Princess Margaret Hospital, Toronto, Ontario, Canada.

## Abstract

Ormaplatin (also known as tetraplatin) is a platinum-containing analogue which has recently undergone clinical trials. Ormaplatin may undergo conversion to dichloro(D,L-trans)-1,2-diaminocyclohexaneplatinum(II) [P1Cl2(trans-dach)]. The cisplatin-resistant murine lymphoma cell lines E8 and E5, were found to be cross-resistant to ormaplatin and PtCl2(trans-dach). We found an inverse rank correlation between drug resistance and drug accumulation for PtCl2(trans-dach) similar to our previous findings with cisplatin; however, accumulation of ormaplatin in the resistant cells was increased. Ormaplatin cytotoxicity appears to result primarily from extracellular conversion to PtCl2(trans-dach), since ormaplatin cytotoxicity was decreased under conditions where extracellular conversion to PtCl2(trans-dach) was minimised. Co-incubation with different inhibitors of energy metabolism resulted in a 65-70% increase in PtCl2(trans-dach) accumulation in the parental cell line R1.1 and a 113-307% increase in the resistant cell line E5 which suggests that the decrease in accumulation in E5 may be at least partly energy dependent. We conclude from these findings that cross-resistance to ormaplatin is associated with an energy-dependent decreased accumulation of PtCl2(trans-dach) in these cisplatin-resistant cell lines.


British Journal of Cancer (1996) 74, 590-596
© 1996 Stockton Press All rights reserved 0007-0920/96 $12.00

### Ormaplatin resistance is associated with decreased accumulation of its platinum (II) analogue, dichloro(D,L-*trans*)1,2-diaminocyclohexaneplatinum(II)

D Rischin* and V Ling**

*The Ontario Cancer Institute, Princess Margaret Hospital and Department of Medical Biophysics, 500 Sherbourne Street, Toronto, Ontario, Canada M4X 1K9.*

**Summary** Ormaplatin (also known as tetraplatin) is a platinum-containing analogue which has recently undergone clinical trials. Ormaplatin may undergo conversion to dichloro(D,L-*trans*)-1,2-diaminocyclohexaneplatinum(II) [$PtCl_2$(*trans*-dach)]. The cisplatin-resistant murine lymphoma cell lines E8 and E5, were found to be cross-resistant to ormaplatin and $PtCl_2$(*trans*-dach). We found an inverse rank correlation between drug resistance and drug accumulation for $PtCl_2$(*trans*-dach) similar to our previous findings with cisplatin; however, accumulation of ormaplatin in the resistant cells was increased. Ormaplatin cytotoxicity appears to result primarily from extracellular conversion to $PtCl_2$(*trans*-dach), since ormaplatin cytotoxicity was decreased under conditions where extracellular conversion to $PtCl_2$(*trans*-dach) was minimised. Co-incubation with different inhibitors of energy metabolism resulted in a 65–70% increase in $PtCl_2$(*trans*-dach) accumulation in the parental cell line R1.1 and a 113–307% increase in the resistant cell line E5 which suggests that the decrease in accumulation in E5 may be at least partly energy dependent. We conclude from these findings that cross-resistance to ormaplatin is associated with an energy-dependent decreased accumulation of $PtCl_2$(*trans*-dach) in these cisplatin-resistant cell lines.

**Keywords:** drug resistance; organoplatinum compound; drug accumulation; neoplasm; metabolic inhibitor

Although *cis*-diamminedichloroplatinum(II) (cisplatin) has significant activity against a broad range of human malignancies, cisplatin-based chemotherapy is frequently not curative. The development of resistance to cisplatin at a cellular level is believed to be a major obstacle to improving the outcome with cisplatin-based chemotherapy. Experimental models of cisplatin resistance have implicated multiple mechanisms including decreased drug accumulation, elevated glutathione and metallothionein levels, increased DNA repair and increased tolerance of DNA damage (Andrews and Howell, 1990; Shellard *et al.*, 1993).

One approach to circumventing cisplatin resistance is the development of platinum-containing analogues that are not cross-resistant. Ormaplatin is one such analogue that was not cross-resistant in the initial studies in L1210 and P388 leukaemias (Anderson *et al.*, 1986; Wilkoff *et al.*, 1987) or in cisplatin-resistant human lung cancer cell lines (Ohmori *et al.*, 1993). Other studies have revealed that although ormaplatin has a different pattern of sensitivity compared with cisplatin or carboplatin, partial cross-resistance to ormaplatin may occur (Hills *et al.*, 1989; Perez *et al.*, 1991; Teicher *et al.*, 1991; Bhuyan *et al.*, 1991; Kelland *et al.*, 1992). The mechanisms of cross-resistance to ormaplatin have not been extensively studied, but may be complex (Parker *et al.*, 1993). Ormaplatin has also been found to be less nephrotoxic than cisplatin (Smith *et al.*, 1988), but neurotoxicity has been a significant problem in early clinical trials (Schilder *et al.*, 1994; O'Rourke *et al.*, 1994).

Ormaplatin is a platinum (IV) compound which can be reduced to the platinum (II) compound dichloro(D,L,-*trans*)-1,2 - diaminocyclohexaneplatinum (II) [$PtCl_2$(*trans* - dach)] (Eastman, 1987; Gibbons *et al.*, 1989) (Figure 1). It has been demonstrated that ormaplatin is converted to $PtCl_2$(*trans*-dach) in both tissue culture media and rat plasma (Gibbons *et al.*, 1989; Chaney *et al.*, 1990). Although it has been thought that this reduction could also occur intracellularly (Eastman, 1987), recent work has revealed unexpected intracellular biotransformation pathways for ormaplatin which do not result in formation of $PtCl_2$(*trans*-dach) (Chaney *et al.*, 1991).

Our laboratory has previously described a series of cisplatin-resistant cell lines (E8 and E5) derived from the murine lymphoma cell line, R1.1. In these cisplatin-resistant cell lines there is an inverse rank correlation between the level of resistance and intracellular drug accumulation (Kawai *et al.*, 1990). The mechanism of decreased accumulation in cisplatin-resistant cell lines is poorly understood. It is also unclear whether cisplatin or other platinum-containing analogues cross the plasma membrane by passive diffusion or by a mediated route. In this paper we demonstrate cross-resistance to ormaplatin in these cell lines, which is associated with decreased accumulation of $PtCl_2$(*trans*-dach) but not ormaplatin. The mechanism of decreased accumulation and the factors affecting the reduction of ormaplatin to $PtCl_2$(*trans*-dach) were also investigated.

### Materials and methods

### *Cell lines*

The murine T cell lymphoma cell line R1.1 and its cisplatin-resistant variants, E8 and E5, have been described previously (Kawai *et al.*, 1990). These resistant cell lines were selected by continuous exposure to cisplatin and exhibit decreased accumulation of cisplatin. All cell lines were grown as monolayers in RPMI 1640 medium (Gibco, Burlington, Ontario, Canada) supplemented with 10% fetal bovine serum (FBS) (Hyclone, Logan, Utah, USA), 100 units $ml^{-1}$ penicillin and 100 $\mu g\ ml^{-1}$ streptomycin sulphate. All cell cultures were incubated at 37°C in a humidified atmosphere containing 95% air and 5% carbon dioxide.

Correspondence: D Rischin, Division of Haematology and Medical Oncology, Peter MacCallum Cancer Institute, Locked Bag No 1, A'Beckett St., Melbourne, Victoria, Australia, 3000
*Present address: Division of Haematology and Medical Oncology, Peter MacCallum Cancer Institute, Locked Bag No 1, A'Beckett St., Melbourne, Victoria, Australia, 3000
**Present address: BC Cancer Research Centre, 601 West 10th Avenue, Vancouver, BC, Canada V5Z IL5
Received 20 December 1995; revised 18 March 1996; accepted 21 March 1996

*Chemicals*

Unlabelled and $^3$H-labelled ormaplatin and $PtCl_2$(*trans*-dach) were generous gifts from Dr R Haugwitz (Drug Synthesis and Chemistry Branch, National Cancer Institute, Bethesda, MD, USA). Clinical formulations of cisplatin and carboplatin were obtained from Bristol Myers-Squibb (Belleville, Ontario, Canada). Cystine and other components of RPMI-medium were obtained from Gibco. Sodium azide, 2,4-dinitrophenol and 5,5′-dithio-bis (2-nitrobenzoic acid) were purchased from Sigma (St Louis, MO, USA). MTT was purchased from ICN Biochemicals (Cleveland, OH, USA). Phosphate-buffered saline (PBS) used in these experiments contained: (in g $l^{-1}$) sodium chloride, 8; potassium chloride, 0.2; potassium hydrogen phosphate, 0.2; sodium hydrogen phosphate, 1.15; calcium chloride hydrate, 0.132; magnesium chloride hexahydrate, 0.1.

*Drug resistance*

The level of drug resistance was examined using the MTT assay as previously described (Kawai *et al.*, 1990; Carmichael *et al.*, 1987) with minor modifications. Briefly, cells (4000–5000) seeded in microtitre plates were allowed to attach for 4 h and then exposed to drug for 1 or 96 h at 37°C. For the 1 h exposure assay, cells were exposed to drug, then medium was aspirated and the wells washed twice with ice-cold PBS. Cells were then incubated in fresh medium for 96 h. After 96 h MTT (50 $\mu$l of 2 mg $ml^{-1}$) was added to each well and plates were incubated for another 4 h. Plates were centrifuged, then wells were aspirated, 100 $\mu$l dimethyl sulphoxide was added to each well and absorbance at a wavelength of 540 nm measured. During the 96 h drug exposures the medium was not changed, and the drug was not washed out before the addition of MTT.

*Drug accumulation*

Cells were seeded in 60 mm tissue culture dishes 2 days before examining drug accumulation. At the time of the experiments cells were in exponential phase and approximately 50% confluent. Medium was aspirated and dishes washed twice with prewarmed PBS before adding transport buffer (phosphate-buffered saline with 11 mM glucose, pH 7.3) containing radiolabelled drug. Incubations of less than 10 min duration took place in a 37°C oven and for longer incubations dishes were returned to the tissue culture incubator (37°C, 5% carbon dioxide). Incubations were terminated by rapidly aspirating buffer and washing three times in ice-cold PBS. Cells were digested in 3 ml of 0.5 M sodium hydroxide overnight. A 10 $\mu$l aliquot was taken for duplicate protein estimation by the method of Bradford modified for microtitre plate assay (Bradford, 1976; Simpson and Sonne, 1982). A 1 ml aliquot was mixed with scintillation fluid (ICN) and 50 $\mu$l glacial acetic acid and counted on a Beckman LS-6000 scintillation counter using the auto-d.p.m. setting. Surface binding was estimated by incubating cells with drug for 15 s at 4°C. This value was subtracted from all accumulation measurements. For exposures of $\geqslant$30 min surface binding was $<$5% of the total accumulation. For 5 min exposures surface binding represented 15% and 25% of the total accumulation for R11 and E5 respectively. In experiments with metabolic inhibitors cells were preincubated in PBS containing inhibitor but no glucose for 5 min and then exposed to buffer without glucose containing both radiolabelled $PtCl_2$(*trans*-dach) and the inhibitor for another 5 min. In efflux studies cells were preloaded with [$^3$H]$PtCl_2$(*trans*-dach) for 15 min. Concentrations of 2.5 $\mu$M for R1.1 and 3.75 $\mu$M for E5 were used to give approximately equal intracellular drug accumulation. Cells were then washed twice with ice-cold PBS and drug-free buffer was added. Remaining intracellular drug concentration was assessed over the next 30 min as described above.

*HPLC analysis of ormaplatin reduction*

HPLC analysis of ormaplatin and $PtCl_2$(*trans*-dach) was performed with a 250 × 4.6 mm Zorbax 7 ODS column (Phenomenex, Rancho Palos Verdes, CA, USA) using water as the mobile phase at a flow rate of 0.5 ml $min^{-1}$ as previously described (Anderson *et al.*, 1986; Gibbons *et al.*, 1989). In the concentration range we were using $PtCl_2$(*trans*-dach) was not detectable with ultraviolet photometric detection (HPLC-UV) as it has much lower absorptivity than ormaplatin at 254 nm (Bhuyan *et al.*, 1991). Therefore, the decrease in the ormaplatin peak on HPLC-UV was routinely used to estimate the percentage reduction of ormaplatin. Simultaneous HPLC analysis using a radiochemical detector revealed that the combined ormaplatin and $PtCl_2$(*trans*-dach) peaks did not decrease under any of the experimental conditions we employed which is consistent with the decreases in the ormaplatin peak detected by HPLC-UV being due to ormaplatin being converted to $PtCl_2$(*trans*-dach). [$^3$H]$PtCl_2$(*trans*-dach) (2.5 $\mu$M) was incubated in various transport buffers in the presence or absence of cells for 30 min at 37°C in the tissue culture incubator. After this incubation an aliquot of the buffer was centrifuged in an air-driven ultracentrifuge (Beckman) for 15 s to remove any cells or debris and then 50 $\mu$l was injected for HPLC analysis.

*Extracellular thiol concentration*

Sulfhydryl content was determined using a modification of the Ellman method (Ellman, 1980). Cells were seeded in 100 mm tissue culture dishes two days before experiments at which time they were in exponential phase. Cells were incubated in 5 ml transport buffer with or without cystine for 1 h at 37°C in the tissue culture incubator. After incubation 1.5 ml of the buffer was removed and centrifuged for 2 min at 2000 r.p.m. in a tabletop centrifuge to remove any floating cells. An aliquot of 1.5 ml of the buffer was mixed with 1.5 ml of 0.2 M potassium phosphate–10 mM EDTA, pH 8.0. Absorbance at 412 nm was measured, then 210 $\mu$l of 6 mM 5,5′-dithiobis (2-nitrobenzoic acid) was added and the increase in absorbance was measured after 5 min. The sulfhydryl content was calculated from the increase in absorbance using cysteine as a standard.

### Results

*Drug resistance*

In the 4 day continuous exposure assays there was partial cross-resistance to ormaplatin but marked cross-resistance to carboplatin, relative to the resistance to cisplatin (Table I). The degree of cross-resistance to the d and l isomers of ormaplatin was similar to the racemic mixture, though the l isomer was less potent than the d isomer. As expected based on the known conversion of ormaplatin to $PtCl_2$(*trans*-dach) in tissue culture media (Gibbons *et al.*, 1989), $PtCl_2$(*trans*-dach) gave very similar results to ormaplatin. In the 1 h exposure assays the fold resistance to cisplatin was less than in the 4 day assays, but the cross-resistance to ormaplatin was greater in the 1 h assay particularly in E5. In a study of cisplatin-resistant human ovarian cell lines, it was proposed that there are two platinum-resistant phenotypes; one with high levels of resistance to cisplatin and carboplatin and more moderate resistance to ormaplatin, and the other with low levels of resistance to cisplatin and carboplatin and comparatively high levels of resistance to ormaplatin (Perez *et al.*, 1991). The drug exposure time may need to be considered when examining resistance to platinum complexes as our platinum-resistant cell line E5 could be classified in either category depending on the duration of drug exposure. For the 1 h exposure ormaplatin was preincubated in RPMI medium with 10% fetal bovine serum before addition to cells. Under these conditions HPLC analysis revealed that over 90% of the ormaplatin was reduced to $PtCl_2$(*trans*-dach)

**Table I** Sensitivity of cell lines to various platinum-containing analogues [$IC_{50}$ ($\mu$M) ± s.e.]

| *Drugs* | *R11* | *E8* | *E5* |
|---|---|---|---|
| Four day exposure | | | |
| Cisplatin | 0.73 ± 0.10[a] (1) | 9.43 ± 0.02 (12.9)[b] | 18.27 ± 1.02 (24.9) |
| Carboplatin | 3.89 ± 0.30 (1) | 82.08 ± 1.30 (21.1) | 152.54 ± 1.70 (39.2) |
| Ormaplatin (d,1) | 0.19 ± 0.02 (1) | 0.75 ± 0.02 (4.0) | 1.57 ± 0.10 (8.3) |
| Ormaplatin (d) | 0.18 ± 0.01 (1) | 0.90 ± 0.02 (5) | 1.94 ± 0.07 (10.8) |
| Ormaplatin (1) | 0. 41 ± 0.01 (1) | 2.26 ± 0.01 (5.51) | 4.48 ± 0.01 (11.9) |
| $PtCl_2$ (*trans*-dach) | 0.15 ± 0.01 (1) | 0.60 ± 0.07 (4.0) | 1.36 ± 0.06 (9.1) |
| One hour exposure | | | |
| Cisplatin | 19.47 ± 1.00 (1) | 97.03 ± 8.50 (5.0) | 234.70 ± 9.7 (12.5) |
| Ormaplatin (d,1)[c] | 0.86 ± 0.05 (1) | 5.22 ± 0.36 (6.1) | 46.64 ± 4.0 (54.2) |
| Ormaplatin (d,1)PBS[d] | 13.82 ± 2.58 (1) | 47.60 ± 6.10 (3.4) | 96.90 ± 11.1 (7.0) |

[a] $IC_{50}$ values were determined by MTT assay and represent the means ± s.e. of 2–4 separate experiments each done in quadruplicate. [b] Figures in parentheses represent the fold resistance relative to R11. [c] Ormaplatin was incubated in RPMI + 10% fetal bovine serum for 1 h at 37°C before addition to cells. [d] Cells exposed to ormaplatin in PBS + glucose.

(data not shown). When ormaplatin was tested in PBS instead of RPMI medium supplemented with 10% fetal bovine serum, in order to minimise reduction of ormaplatin, the $IC_{50}$s were considerably higher particularly in R1.1 and E8. While the cisplatin-resistant cells were still cross-resistant to ormaplatin in PBS, the degree of cross-resistance was less than that observed in RPMI medium with serum.

### *Drug accumulation*

In preliminary experiments it was noted that when R1.1 and E5 cells were exposed to radiolabelled ormaplatin the intracellular accumulation of radiolabelled drug was decreased in RPMI medium without serum compared with PBS. Furthermore, there was less drug accumulated in the resistant cells compared with the parental cells when incubated in medium but this was not the case when the incubation took place in PBS. In PBS there was in fact a trend for increased drug accumulation in the resistant cell lines (Figure 2). This was confirmed by experiments in which accumulation of ormaplatin in PBS over 5 min revealed a 28–66% increase in E5 in the 1–10 $\mu$M concentration range (data not shown).

In order to determine which component of RPMI medium modulated the accumulation of ormaplatin, accumulation was measured in different PBS buffers each supplemented with a component of RPMI medium. Only the addition of the amino acid cystine resulted in decreased accumulation of radiolabelled drug similar to that seen with complete RPMI medium. When cells were exposed to ormaplatin the accumulation of radiolabelled drug in the presence of PBS supplemented with cystine was similar to the accumulation of $PtCl_2$(*trans*-dach), with an inverse rank correlation with the level of resistance (Figure 2). This raised the possibility that in the presence of cystine, ormaplatin was being converted to $PtCl_2$(*trans*-dach), although previous work had suggested that cystine was not one of the components of RPMI medium which could convert ormaplatin to $PtCl_2$(*trans*-dach) (Gibbons *et al.*, 1989).

### *Reduction of ormaplatin in transport buffer*

In order to determine the fate of ormaplatin in different transport buffers HPLC analysis was performed. The percentage of ormaplatin remaining at 30 min was calculated (Table II). There was little conversion to $PtCl_2$(*trans*-dach) in PBS and glucose but in the presence of cells there was an unexpected 40–50% reduction. The addition of cystine resulted in a 40% decrease and in the presence of R1.1 and E5 cells there was a 64% and 91% decrease respectively. The effect of cystine was also demonstrated by the fact that in RPMI medium the reduction of ormaplatin was approximately 20% greater than in RPMI medium without cystine. The most extensive reduction of ormaplatin in the absence of cells took place in RMPI medium supplemented with 10% fetal bovine serum.

Ormaplatin

$PtCl_2$(*trans*-dach)

**Figure 1** Structures of ormaplatin and dichloro(D,L-*trans*)1,2-diaminocyclohexaneplatinum(II) [$PtCl_2$(*trans*-dach)].

**Figure 2** Accumulation of ormaplatin and $PtCl_2$(*trans*-dach) in R1.1 and its cisplatin-resistant variants E8 and E5. Thirty min accumulation of 2.5 $\mu$M ormaplatin (open bars, in PBS + glucose; solid bars, in PBS + glucose + cystine) and 2.5 $\mu$M $PtCl_2$(*trans*-dach) (hatched bars, in PBS + glucose). Results are the means ± s.d. of three experiments.

### *Extracellular thiol concentration*

As the reduction of ormaplatin in PBS and cystine was significantly greater in the presence of E5 cells than R1.1 cells

**Table II** Reduction of ormaplatin to $PtCl_2$ (*trans*-dach) in different transport buffers as determined by reversed-phase HPLC analysis

| *Transport buffer* | *Percentage of ormaplatin remaining after 30 min incubation (mean ± s.d.; n = 3)* |
|---|---|
| PBS + glucose | 93.9 ± 6.9 |
| PBS + glucose, incubating R1.1 cells | 58.6 ± 4.9 |
| PBS + glucose, incubating E5 cells | 51.7 ± 7.9 |
| PBS + glucose + cystine | 61.0 ± 13.6 |
| PBS + glucose + cystine, incubating R1.1 | 33.8 ± 3.3 |
| PBS + glucose + cystine incubating E5 | 9.3 ± 4.0 |
| RPMI medium | 49.3 ± 5.2 |
| RPMI medium without cystine | 68.8 ± 7.4 |
| RPMI medium + 10% FBS | 32.0 ± 5.2 |

we sought to determine whether there was a difference in the formation of extracellular thiols that could account for it. Thiols in media supplemented with serum and in rat plasma have previously been found to be important reducing agents for the conversion of ormaplatin to $PtCl_2$(*trans*-dach) (Gibbons *et al.*, 1989; Chaney *et al.*, 1990). However, the role of thiols which have effluxed out of cells has not been previously determined. The extracellular thiol concentration was 1.4 ± 0.3 μM following incubation of R1.1 in PBS and 2.4 ± 1.5 μM following incubation of the cells in PBS containing cystine. Following incubation of E5 cells the respective values were 2.1 ± 0.8 μM in PBS and 39.7 ± 6.5 μM in PBS containing cystine. The marked increase in extracellular thiol formation in the presence of E5 compared with R1.1 when cystine was present would therefore explain the more rapid reduction of ormaplatin in the presence of E5 cells.

*Transport of* $PtCl_2$(trans-*dach*)

As accumulation of $PtCl_2$(*trans*-dach) or ormaplatin under conditions in which it was predominantly converted to $PtCl_2$(*trans*-dach), was reduced in the platinum-resistant cells the transport of $PtCl_2$(*trans*-dach) was studied in more detail. The accumulation of 2.5 μM $PtCl_2$(*trans*-dach) in E5 was 37–53% less than in R1.1 over the first hour (Figure 3). From 2.5 up to 100 μM there is a 40–50% reduction in the 5 min accumulation of $PtCl_2$(*trans*-dach) in E5 (Figure 4). The increase in accumulation with increasing concentration is linear in R1.1. In E5 there is some deviation from linearity at 100 μM but the low solubility of this compound prevented testing at higher concentrations to determine whether there really was saturation. Although these studies were done over a relatively short time interval (5 min), they do not necessarily represent zero-*trans* conditions which are ideally required to measure drug influx. However, efflux studies demonstrated that there was no increase in efflux in E5 that could account for the lower drug accumulation in these cells (Figure 5). Hence these results are more consistent with decreased influx rather than increased efflux.

*Effect of temperature, sodium, metabolic inhibitors and platinum-containing analogues on the accumulation of* $PtCl_2$(trans-*dach*)

The 5 min accumulation of 2.5 μM [$^3$H]$PtCl_2$(*trans*-dach) was measured under various conditions and the results expressed as a percentage of the accumulation of the drug in R1.1 or E5 at 37°C (Table III). The accumulation was temperature dependent with an 80–84% decrease at 4°C. The absence of sodium in the transport buffer resulted in only a very minor decrease in accumulation. The effect of metabolic inhibitors was most intriguing. Both sodium azide and dinitrophenol resulted in a 65–70% increase in drug accumulation in R1.1. In E5 the effect was even more dramatic with a 113% increase with sodium azide and a 307% increase with

**Figure 3** Accumulation of 2.5 μM $PtCl_2$(*trans*-dach) over 60 min in R1.1 (–■–) and E5 (- -O- -). Points are the means ± s.d. of three experiments. When the s.d. was less than the symbol size, error bars were omitted.

**Figure 4** Accumulation of $PtCl_2$(*trans*-dach) from 2.5 μM up to 100 μM in R1.1 (–■–) and E5 (- -O- -). Accumulation at 5 min was measured. Inset shows accumulation in lower concentration range. Points are the means ± s.d. of three experiments. When the s.d. was less than the symbol size, error bars were omitted.

dinitrophenol. In order to exclude a generalised increase in permeability with these inhibitors that could account for the increased accumulation, the permeability to trypan blue was assessed. We found no difference in the permeability of R1.1 or E5 to trypan blue in the presence or absence of the inhibitors after 10 or 30 min incubations. One explanation for such an increase in accumulation under conditions of energy depletion would be the presence of an energy-dependent efflux pump. We measured the efflux of drug in the presence or absence of sodium azide. At 5 min the percentage of drug remaining intracellularly in the absence of sodium azide was 83.7% ± 12.7 in R11 and 87.2% ± 9.1 in E5. In the presence of 10 mM sodium azide the corresponding values were 88.8% ± 8.0 in R11 and 75.7% ± 7.2 in E5. The differences in efflux in the presence or absence of sodium azide were not significant and there was certainly no trend for a decrease in efflux in the presence of azide.

A 40-fold excess of unlabelled $PtCl_2$(*trans*-dach) resulted in a 40% decrease in the accumulation of radiolabelled drug, which would not be expected if the entry into cells of $PtCl_2$(*trans*-dach) was by passive diffusion alone. Neither cisplatin nor transplatin had any effect on $PtCl_2$(*trans*-dach)

accumulation. Interestingly, excess ormaplatin appeared to stimulate the accumulation of $PtCl_2$(*trans*-dach), with a more pronounced effect in E5. Accumulation of $PtCl_2$(*trans*-dach) after preincubation with excess ormaplatin was not increased.

### Discussion

Our studies have demonstrated that the E8 and E5 cisplatin-resistant cell lines are cross-resistant to ormaplatin and $PtCl_2$(*trans*-dach). Furthermore, these cell lines which have previously been demonstrated to have decreased cisplatin accumulation, also exhibit energy-dependent decreased accumulation of $PtCl_2$(*trans*-dach), a drug to which they have not been previously exposed. Our cytotoxicity, HPLC and drug accumulation data are consistent with ormaplatin being a prodrug for $PtCl_2$(*trans*-dach) as has been proposed by other investigators (Anderson *et al.*, 1986; Gibbons *et al.*, 1989).

The cytotoxicity of ormaplatin in PBS was much less than in RPMI medium with 10% fetal bovine serum. Our HPLC analysis demonstrated that even in PBS in the presence of cells there is significant reduction of ormaplatin. The

**Figure 5** Efflux of $PtCl_2$(*trans*-dach) from R1.1 (-■-) and E5 (- -O- -). R1.1 was loaded with 2.5 μM [$^3$H]$PtCl_2$(*trans*-dach) and E5 with a 3.75 μM, each for 15 min. Release of drug into drug-free buffer was measured over 30 min. Points are the means ± s.d. of three experiments. When the s.d. was less than the symbol size, error bars were omitted.

cytotoxicity that is seen in PBS could be largely owing to the $PtCl_2$(*trans*-dach) that was formed in the extracellular buffer, and it is less than in media with serum as there is less $PtCl_2$(*trans*-dach) formed in PBS. These results suggest that the ormaplatin that crosses the plasma membrane without prior reduction is less cytotoxic than ormaplatin which is reduced to $PtCl_2$(*trans*-dach) extracellularly. In fact ormaplatin that is not reduced extracellularly may not result in any significant cytotoxicity, at least in the cell lines we have examined. These findings are consistent with the findings of Chaney and colleagues who demonstrated in an L1210 cell line that most of the ormaplatin was not converted to $PtCl_2$(*trans*-dach) intracellularly but to two other transformation products that were not reactive with DNA (Chaney *et al.*, 1991). Our results differ from the report by Eastman in an L1210 cell line where he found increased ormaplatin cytotoxicity in Hanks' balanced salt solution, and postulated that ormaplatin could be reduced to $PtCl_2$(*trans*-dach) intracellularly (Eastman, 1987). In another study using the same L1210 cell lines the cytotoxicity of ormaplatin in Hanks' balanced salt solution was increased by the addition of glutathione which increased extracellular conversion to $PtCl_2$(*trans*-dach) (Kido *et al.*, 1994). All these studies support the hypothesis that ormaplatin requires reduction to $PtCl_2$(*trans*-dach) for biological activity.

Our HPLC results emphasise the importance of determining the amount of ormaplatin reduction that occurs under the conditions used in cytotoxicity or transport studies. It had not been previously appreciated that there would be an increase in the reduction of ormaplatin in the presence of cells, nor that cystine in the media may contribute to this reduction of ormaplatin. The increased formation of thiols in the extracellular buffer in the presence of cystine is probably due to the uptake of cystine by the cells, its rapid conversion to cysteine and subsequent efflux out of the cells, as has been demonstrated in skin fibroblasts (Bannai and Ishii, 1980). The striking difference in the concentration of extracellular thiols between sensitive and resistant cells can be at least partly explained by the fact that there is increased cystine uptake in the resistant cells (D Rischin and V Ling, manuscript in preparation). Previous reports of the percentage of ormaplatin remaining after a 30 min incubation in RPMI medium with 15% fetal bovine serum have ranged from 5% to over 80% (Bhuyan *et al.*, 1991; Gibbons *et al.*, 1989). Our result of 32% was obtained with a lower percentage of fetal bovine serum, 10%. These variable results may be caused by different sources of serum.

Similar to our previous findings with cisplatin (Kawai *et al.*, 1990) there was an inverse rank correlation between the level of ormaplatin or $PtCl_2$(*trans*-dach) resistance and the accumulation of $PtCl_2$(*trans*-dach), suggesting that decreased

**Table III** Effect of temperature, sodium, metabolic inhibitors and other platinum-containing analogues on the accumulation of 2.5 μM [$^3$H]$PtCl_2$ (*trans*-dach)

| Conditions | *Percentage of control accumulation ± s.d. (n = 3–6)* *R11* | *E5* |
|---|---|---|
| 4°C | 16.0 ± 8.9 | 20.7 ± 2.5 |
| Sodium-free buffer[a] | 93.5 ± 2.5 | 89.0 ± 8.2 |
| Sodium azide (10 mM)[b] | 169.2 ± 33.4 | 212.7 ± 21 |
| Dinitrophenol (1 mM)[b] | 165.7 ± 26.8 | 406.7 ± 86.1 |
| $PtCl_2$ (*trans*-dach) (100 μM)[c] | 62.0 ± 4.1 | 59.0 ± 12.5 |
| Cisplatin (100 μM)[c] | 103.0 ± 6.0 | NT[d] |
| Transplatin (100 μM)[c] | 97.4 ± 7.5 | NT |
| Ormaplatin (100 μM)[c] | 125.3 ± 3.1 | 164.7 ± 20.0 |
| Preincubation ormaplatin (100 μM)[e] | 113.7 ± 22.3 | 105.0 ± 5.3 |

Five minute accumulation of 2.5 μM [$^3$H]$PtCl_2$ (*trans*-dach) was measured under various conditions. Results are expressed as a percentage of the accumulation of 2.5 μM [$^3$H]$PtCl_2$ (*trans*-dach) in R11 or E5 at 37°C in PBS without any added drugs. [a] Sodium was replaced by choline. [b] Cells were preincubated with inhibitor for 5 min, then exposed to drug with inhibitor for 5 min. [c] Co-incubation of [$^3$H]$PtCl_2$ (*trans*-dach) with 40-fold excess of unlabelled drug. [d] NT, not tested. [e] Preincubation for 10 min then cells exposed to 2.5 μM $PtCl_2$ (*trans*-dach) alone.

accumulation may play a role in the cross-resistance to these drugs in the platinum-resistant cell lines. However, it cannot fully explain the dramatic increase in ormaplatin resistance between E8 and E5 seen in the 1 h exposure assays. Ormaplatin accumulation was actually increased in the resistant cells which provides further indirect support for the notion that $PtCl_2$(*trans*-dach) rather than ormaplatin accumulation contributes to ormaplatin sensitivity.

Studies in L1210 models have demonstrated that the cisplatin-resistant variants have only minor levels of cross-resistance to ormaplatin or $PtCl_2$(*trans*-dach) (Richon *et al.*, 1987; Kraker and Moore, 1988; Goddard *et al.*, 1991; Nicolson *et al.*, 1992), which in turn is associated with only slight decreases in the accumulation of $PtCl_2$(*trans*-dach) (Richon *et al.*, 1987; Nicholson *et al.*, 1992; Gibbons *et al.*, 1990). However, other cisplatin-resistant variants have more significant levels of cross-resistance to ormaplatin (Teicher *et al.*, 1991; Bhuyan *et al.*, 1991; Kelland *et al.*, 1992). L1210 variants selected in $PtCl_2$(*trans*-dach) or ormaplatin have demonstrated significantly decreased accumulation of $PtCl_2$(*trans*-dach) (Richon *et al.*, 1987; Nicolson *et al.*, 1992; Gibbons *et al.*, 1990). The mechanism of decreased accumulation of $PtCl_2$(*trans*-dach) has not been studied extensively. One recent study found no difference in efflux (Nicolson *et al.*, 1992). We also found that there was no apparent increase in efflux in the resistant cells. The decrease in accumulation was detectable at low concentrations and over short time intervals (2–5 min). Most interestingly, inhibition of energy metabolism resulted in increased accumulation with a more marked effect in the resistant cells. There did not appear to be any decrease in efflux in the presence of sodium azide, as would be expected if there was an energy-dependent efflux transporter involved. Our results would appear to favour decreased influx as the mechanism of decreased accumulation. One possibility is that there is an energy-dependent restriction to $PtCl_2$(*trans*-dach) influx which is greater in the resistant cells, so that in the absence of energy the accumulation between the sensitive and the resistant cells is decreased or abolished. However, as increased accumulation following inhibition of energy metabolism is more characteristic of an efflux transporter, the possibility of increased efflux in the resistant cells cannot be excluded despite the fact that we could not demonstrate any such increase. It is interesting to note that in some multidrug-resistant cell lines that overexpress P-glycoprotein reduced influx has been reported (Sirotnak *et al.*, 1986; Ramu *et al.*, 1989). It has been postulated that the influx of drug can be altered by the presence of an efflux pump such as P-glycoprotein under certain circumstances (Demant *et al.*, 1990). The effect of energy depletion on $PtCl_2$(*trans*-dach) transport has not been previously reported. In our cell lines neither sodium azide nor dinitrophenol altered cisplatin accumulation (data not shown). Conflicting results have been reported in studies examining the effect of energy depletion on cisplatin accumulation in human ovarian carcinoma cell lines. Andrews *et al.* (1988) found that a combination of inhibitors of oxidative phosphorylation and inhibitors of glycolysis resulted in a decrease in cisplatin accumulation in both sensitive and resistant cells, while Sharp *et al.* (1995) demonstrated an increase in cisplatin accumulation in both sensitive and resistant cells under similar conditions.

As to whether there is a mediated component to $PtCl_2$(*trans*-dach) influx we did not demonstrate saturation but we were unable to go above 100 $\mu$M owing to the low solubility of $PtCl_2$(*trans*-dach). The accumulation of $PtCl_2$(*trans*-dach) was inhibited by unlabelled drug and was temperature dependent, features which would be consistent with mediated transport but in themselves do not establish its presence. It seems unlikely that the decreased accumulation of $PtCl_2$(*trans*-dach) and cisplatin in these resistant cell lines share a common mechanism, as there was no inhibition of $PtCl_2$(*trans*-dach) accumulation by excess cisplatin, and $PtCl_2$(*trans*-dach) accumulation but not cisplatin accumulation is energy dependent. Our laboratory has been interested in identifying plasma membrane proteins that are differentially expressed between cisplatin-sensitive and -resistant cells hypothesising that such a protein or proteins may play a role in mediating the decreased accumulation of cisplatin and/or $PtCl_2$(*trans*-dach) in the resistant cells. The more marked effect of metabolic inhibitors on $PtCl_2$(*trans*-dach) accumulation in resistant cells than sensitive cells would be consistent with the presence of an energy-dependent transporter with altered expression in the resistant cells. We have previously reported the overexpression of a plasma membrane glycoprotein $CP^R$-200 in the cisplatin-resistant variants of R1.1 (Kawai *et al.*, 1990), while others have reported decreased expression of the membrane protein SQM1 in platinum-resistant human squamous carcinoma cell lines (Bernal *et al.*, 1990) and the overexpression of a 36 kDa plasma membrane protein in a cisplatin-resistant human ovarian carcinoma cell line (Sharp *et al.*, 1995). It is not known at the present time whether any of these membrane proteins have a role in mediating the decreased accumulation of platinum that is commonly present in cisplatin-resistant cell lines.

In summary our cisplatin-resistant cell lines are cross-resistant to ormaplatin and $PtCl_2$(*trans*-dach) and this is associated with an energy-dependent decreased accumulation of $PtCl_2$(*trans*-dach). Ormaplatin cytotoxicity appears to result primarily from extracellular conversion to $PtCl_2$(*trans*-dach), since ormaplatin cytotoxicity was decreased under conditions where extracellular conversion to $PtCl_2$(*trans*-dach) was minimised. In plasma rapid conversion of ormaplatin to $PtCl_2$(*trans*-dach) occurs but when ormaplatin is given by other routes, e.g. intraperitoneally (Plaxe *et al.*, 1993), the rate of extracellular conversion to $PtCl_2$(*trans*-dach) may determine the intracellular levels of biologically active drug.

### Abbreviations cisplatin, *cis*-diamminedichloroplatinum(II); ormaplatin, tetrachloro(D,L-*trans*)-1,2-diaminocyclohexaneplatinum(II), also called tetraplatin; $PtCl_2$(*trans*-dach), dichloro(D,L-*trans*)-1,2-diaminocyclohexaneplatinum(II); MTT, 3-(4,5-dimethylthiazol-2-yl)-2,5-diphenyltetrazolium bromide; PBS, phosphate-buffered saline; HPLC, high-pressure liquid chromatography.

### Acknowledgements

Dr Rischin was funded by the George Knudson Fellowship in Cancer Research. We would like to thank Dr A Varghese for assistance with the HPLC assays, and Dr R Haugwitz (Drug Synthesis and Chemistry Branch, National Cancer Institute, Bethesda, MD, USA) for supplying unlabelled and radiolabelled platinum compounds. We would also like to thank Dr T Ohkubo and Dr S Chaney for helpful discussions.

### References

ANDERSON WK, QUAGLIATO DA, HAUGWITZ RD, NARAYANANM VL AND WOLPERT-DEFILLIPES MK. (1986). Synthesis, physical properties, and antitumour activity of tetraplatin and related tetrachloroplatinum(IV) stereoisomers of 1,2-diaminocyclohexane. *Cancer Treat. Rep.*, **70**, 997–1002.

ANDREWS PA AND HOWELL SB. (1990). Cellular pharmacology of cisplatin: Perspectives on mechanisms of acquired resistance. *Cancer Cells*, **2**, 35–43.

ANDREWS PA, VELURY S, MANN SC AND HOWELL SB. (1988). *cis*-diamminedichloroplatinum(II) accumulation in sensitive and resistant human ovarian carcinoma cells. *Cancer Res.*, **48**, 68–73.
BANNAI S AND ISHII T. (1980). Formation of sulfhydryl groups in the culture medium by human diploid fibroblasts. Formation of sulfhydryl groups in the culture medium by human diploid fibroblasts in culture. *J. Cell. Physiol.*, **104**, 215–223.
BERNAL SD, SPEAK JA, BOEHEIM K, DREYFUSS AL, WRIGHT JE, TEICHER BA, ROSOWSKY A, TSAO S-W AND WONG Y-C. (1990). Reduced membrane protein associated with resistance of human squamous carcinoma cells to methotrexate and *cis*-platinum. *Mol. Cell. Biochem.*, **95**, 61–70.
BHUYAN BK, FOIZ SJ, DEZWAAN J, NORTHCOTT SE, ALBERTS DS, GARCIA D, WALLACE TL AND LI LH. (1991). Cytotoxicity of tetraplatin and cisplatin for human and rodent cell lines cultured as monolayers and multicellular spheroids. *Cancer Commun.*, **3**, 53–59.
BRADFORD MM. (1976). A rapid and sensitive method for the quantitation of microgram quantities of protein using the principle of protein-dye binding. *Anal. Biochem.*, **72**, 248–254.
CARMICHAEL J, DEGRAFF W, GAZDAR W, MINNA A AND MITCHELL J. (1987). Evaluation of a tetrazolium-based semi-automatic colorimetric assay: assessment of chemosensitivity testing. *Cancer Res.*, **47**, 936–942.
CHANEY SG, WYRICK S AND TILL GK. (1990). *In vitro* biotransformations of tetrachloro(d,l-*trans*)1,2-diaminocyclohexaneplatinum(IV)(tetraplatin) in rat plasma. *Cancer Res.*, **50**, 4539–4545.
CHANEY SG, GIBBONS GR, WYRICK SD AND PODHASKY P. (1991). An unexpected pathway for tetrachloro-(d,l-*trans*)-1,diaminocyclohexaneplatinum(IV)(tetraplatin) in the L1210 cell line. *Cancer Res.*, **51**, 969–973.
DEMANT EJF, SEHESTED M AND JENSEN PB. (1990). A model for computer simulation of p-glycoprotein and transmembrane pH-mediated anthracycline transport in multidrug-resistant tumor cells. *Biochim. Biophys. Acta*, **1055**, 117–125.
EASTMAN A. (1987). Glutathione-mediated activation of anticancer platinum(IV) complexes. *Biochem. Pharmacol.*, **36**, 4177–4178.
ELLMAN GL. (1959). Tissue sulfhydryl groups. *Arch. Biochem. Biophys.*, **82**, 70–77.
GIBBONS GR, WYRICK S AND CHANEY SG. (1989). Rapid reduction of tetrachloro(D,L-*trans*)1,2-diaminocyclohexaneplatinum(IV) (tetraplatin) in RPMI 1640 tissue culture medium. *Cancer Res.*, **49**, 1402–1407.
GIBBONS GR, PAGE JD, MAUDLIN SK, HUSAIN I AND CHANEY SG. (1990). Role of carrier ligand in platinum resistance in L1210 cells. *Cancer Res.*, **50**, 6497–6501.
GODDARD PM, VALENTI MR AND HARRAP KR. (1991). The role of murine tumour models and their acquired platinum-resistant counterparts in the evaluation of novel platinum antitumour agents: A cautionary note. *Ann. Oncol.*, **2**, 535–540.
HILLS CA, KELLAND LR, ABEL G, SIRACKY J, WILSON AP AND HARRAP KR. (1989). Biological properties of ten human ovarian carcinoma cell lines; calibration *in vitro* against four platinum complexes. *Br. J. Cancer*, **59**, 527–534.
KAWAI K, KAMATANI N, GEORGES E AND LING V. (1990). Identification of a membrane glycoprotein overexpressed in murine lymphoma sublines resistant to *cis*-diamminedichloroplatinum(II). *J. Biol. Chem.*, **265**, 13137–13142.
KELLAND LR, MISTRY P, ABEL G, LOH SY, O'NEILL CF, MURRER BA AND HARRAP KR. (1992). Mechanism-related circumvention of acquired *cis*-diaminedichloroplatinum(II) resistance using two pairs of human ovarian carcinoma cell lines by ammine/amine platinum(IV) dicarboxylates. *Cancer Res.*, **52**, 3857–3864.
KIDO Y, KHOKHAR AR AND SIDDIK ZH. (1994). Glutathione-mediated modulation of tetraplatin activity against sensitive and resistant tumor cells. *Biochem. Pharmacol.*, **47**, 1635–1642.
KRAKER AJ AND MOORE CW. (1988). Accumulation of *cis*-diamminedichloroplatinum(II) and platinum-containing analogues by platinum-resistant murine leukaemic cells *in vitro*. *Cancer Res.*. **48**. 9–13.
NICOLSON MC, ORR RM, O'NEILL CF AND HARRAP KR. (1992). The role of platinum uptake and glutathione levels in L1210 cells sensitive and resistant to cisplatin, tetraplatin or carboplatin. *Neoplasma*, **39**, 3, 189–195.
OHMORI T, MORIKAGE T, SUGIMOTO Y, FUJIWARA Y, KASAHARA K, NISHIO K, OHTA S, SASAKI Y, TAKAHASHI T AND SAIJO N. (1993). The mechanism of the difference in cellular uptake of platinum derivatives in non-small-cell lung cancer cell line (PC-14) and its cisplatin-resistant subline (PC-14/CDDP). *Jpn. J. Cancer* Res., **84**, 83–92.
O'ROURKE TJ, WEISS GR, NEW P, BURRIS III HA, RODRIGUEZ G, ECKHARDT J, HARDY J, KUHN JG, FIELDS S, CLARK GM AND VON HOFF DD. (1994). Phase I clinical trial of ormaplatin (tetraplatin, NSC363812). *Anti-Cancer Drugs*, **5**, 520–526.
PARKER RJ, VIONNET JA, BOSTICK-BRUTON F AND REED E. (1993). Ormaplatin sensitivity/resistance in human ovarian cancer cells made resistant to cisplatin. *Cancer Res.*, **53**, 242–247.
PEREZ RP, O'DWYER PJ, HANDEL LM, OZOLS RF AND HAMILTON TC. (1991). Comparitive cytotoxicity of CI-973, cisplatin, carboplatin and tetraplatin in human ovarian carcinoma cell lines. *Int. J. Cancer*, **48**, 265–269.
PLAXE SC, BRALY PS, FREDDO JL, MCCLAY E, CHRISTEN RD, KIRMANI S, KIM S, HEATH D AND HOWELL SB. (1993). Phase I and pharmacokinetic study of intraperitoneal ormaplatin. *Gynecol. Oncol.*, **51**, 72–77.
RAMU A, POLLARD HB AND ROSARIO LM. (1989). Doxorubicin resistance in P388 leukaemia – evidence for reduced drug influx. *Int. J. Cancer*, **4**, 539–547.
RICHON VM, SCHULTE N AND EASTMAN A. (1987). Multiple mechanisms of resistance to *cis*-diamminedichloroplatinum(II) in murine leukaemia L1210 cells. *Cancer Res.*, **47**, 2056–2061.
SCHILDER RJ, LACRETA FP, PEREZ RP, JOHNSON SW, BRENNAN JM, ROGATKO A, NASH S, MCALEER C, HAMILTON TC, ROBY D, YOUNG RG, OZOLS RF AND O'DWYER PJ. (1994). Phase I and pharmacokinetic study of ormaplatin administered on a day 1 and 8 schedule. *Cancer Res.*, **54**, 709–717.
SHARP SY, ROGERS PM AND KELLAND LR. (1995). Transport of cisplatin and bis-acetato-ammine-dichlorocyclohexyl-amine platinum(IV) (JM216) in human ovarian carcinoma cell lines: identification of a plasma membrane protein associated with cisplatin resistance. *Clin. Cancer Res.*, **1**, 981–989.
SHELLARD SA, FICHTINGER-SCHEPMAN AMJ, LAZO JS AND HILL BT. (1993). Evidence of differential cisplatin–DNA adduct formation, removal and tolerance of DNA damage in three human lung carcinoma cell lines. *Anti-Cancer Drugs*, **4**, 491–500.
SIMPSON IA AND SONNE O. (1982). A simple rapid, and sensitive method for measuring protein concentration in subcellular membrane fractions prepared by sucrose density ultracentrifugation. *Anal. Biochem.*, **119**, 424–427.
SIROTNAK FM, YANG CH, MINES LS, ORIBE E AND BIEDLER J. (1986). Markedly altered membrane transport and intracellular binding of vincristine in multidrug-resistant Chinese hamster cells selected for resistance to vinca alkaloids. *J. Cell. Physiol.*, **126**, 266–274.
SMITH JH, SMITH MA, LITTERST CL, COPLEY MP, UOZUMI J AND BOYD MR. (1988). Comparitive toxicity and renal distribution of the plasma analogs tetraplatin, CHIP, and cisplatin at equimolar doses in the Fisher 344-rat. *Fundam. Applic. Toxicol.*, **10**, 45–61.
TEICHER BA, HOLDEN SA, HERMAN TS, SOTOMAYOR EA, KHANDEKAR V, ROSBE KW, BRANN TW, KORBUT TT AND FREI III E. (1991). Characteristics of five human tumour cell lines and sublines resistant to *cis*-diamminedichloroplatinum(II). *Int. J. Cancer*, **47**, 252–260.
WILKOFF LJ, DULMADGE EA, TRADER MW, HARRISON JR SD AND GRISWOLD JR DP. (1987). Evaluation of *trans*-tetrachloro-1,2-diaminocyclohexane platinum (IV) in murine leukaemia L1210 resistant and sensitive to *cis*-diamminedichloroplatinum (II). *Cancer Chemother. Pharmacol.*, **20**, 96–100.